# Adaptive Evolution of the *Eda* Gene and Scales Loss in Schizothoracine Fishes in Response to Uplift of the Tibetan Plateau

**DOI:** 10.3390/ijms19102953

**Published:** 2018-09-27

**Authors:** Cunfang Zhang, Chao Tong, Arne Ludwig, Yongtao Tang, Sijia Liu, Renyi Zhang, Chenguang Feng, Guogang Li, Zuogang Peng, Kai Zhao

**Affiliations:** 1 Key Laboratory of Adaptation and Evolution of Plateau Biota, Northwest Institute of Plateau Biology, Chinese Academy of Sciences, Xining 810001, China; cfzhang@nwipb.cas.cn (C.Z.); tongchao_2009@sina.com (C.T.); xiaomitang@126.com (Y.T.); sjliu@nwipb.cas.cn (S.L.); zhangrenyi456@163.com (R.Z.); fcg1989@126.com (C.F.); qhnulgg@126.com (G.L.); 2 State Key Laboratory of Plateau Ecology and Agriculture, Qinghai University, Xining 810016, China; 3 Qinghai Province Key Laboratory of Animal Ecological Genomics, Northwest Institute of Plateau Biology, Chinese Academy of Sciences, Xining 810001, China; 4 Department of Evolutionary Genetics, Leibniz Institute for Zoo and Wildlife Research, 10324 Berlin, Germany; ludwig@izw-berlin.de; 5 Key Laboratory of Freshwater Fish Reproduction and Development (Ministry of Education), Southwest University School of Life Sciences, Chongqing 400715, China

**Keywords:** Tibetan plateau, Schizothoracine, scale loss, *Eda* gene, adaptive evolution

## Abstract

Schizothoracine is the predominant wild fish subfamily of the Tibetan plateau (TP). Their scales, pharyngeal teeth and barbels have gradually regressed with increasing altitude. Schizothoracine have been divided into three groups: primitive, specialized and highly specialized. *Ectodysplasin-A* (*Eda*) has been considered as a major gene that contributes to the development of skin appendages. The present study cloned the *Eda* genes of 51 Schizothoracine fish species which represent the three groups and five Barbinae species. Phylogenetic analyses indicated that *Eda* may have acted as the genetic trigger for scale loss in the Schizothoracine. Furthermore, 14 single nucleotide polymorphisms (SNPs) and two deletions (18 bp and 6 bp in size), were also detected in the *Eda* coding sequence of the highly specialized group compared to the primitive group. The same SNPs and two indels result in four non-synonymous and two G-X-Y and 1 XY motif indels, which possibly contribute to significant structure changes in the *Eda* gene. The domain including (G-X-Y)_n_ motif in the *Eda* gene is relatively conserved amongst teleosts. Based on the above results, we hypothesize that the evolution of *Eda* gene might be associated with the scale loss in Schizothoracine fishes in response to the phased uplift of the TP.

## 1. Introduction

The uplift of the Tibetan plateau (TP) is a major historical episode associated with the Earth’s evolutionary history. Research into TP uplift is extremely important for studies on geomorphic development, tectonic activity, and Pleistocene glaciations. Evidences suggest that the three phased uplifts of TP in the late Tertiary period resulted in three marked environmental changes in history, causing pronounced upheaval of the highland [1,2,3]. The uplift induced an increase in TP altitude, and altered the natural conditions of the local environment substantially, such as (i) the colder weather, (ii) intensified radiation, (iii) increased evaporation, and (iv) lower rainfall. In addition, native organisms underwent dramatic selection and extinction [4]. As a result of long-term adaptation, animal morphology has been significantly altered [4]. For example, the body sizes of *Nanorana parkeri* (high Himalayan frog) [5], *Phrynocephalus vlangalii* (Qinghai toad-headed lizard) [6], *Tetraogallus tibetanus* (Tibetan snow cock) [7,8], and *Ochotona curzoniae* (Plateau pika) [9] decreased in conjunction with the increasing altitude. A significant reduction in the size of external organs such as wings in locust [10], scales, pharyngeal teeth and tentacles in fish [11,12], heads, tails and limbs in lizards [13], and external and internal ears in mammals [14,15] also occurred. Schizothoracine fish are widely distributed on the TP, and several research groups have reported the relationship between the origin and evolution of Schizothoracine fish and the uplift of TP [11,16,17,18,19,20,21]. Following phenotypic traits and environmental conditions, Cao et al. [11] found that Schizothoracine subfamily could be divided into three groups: primitive (PG), specialized (SG) and highly specialized (HSG). Each group represents a specific historical stage associated with the phased uplift of TP. The PG which consists of two genera (*Schizothorax* and *Aspiorhynchus)* and including 42 species and subspecies, live in a low elevation environment (1250–2500 m), and possess complete scales, two pairs of pharyngeal teeth and barbels. The SG consists of three genera (*Diptychus, Ptychobarbus* and *Gymnodiptychus*), including nine species and subspecies. They are predominantly distributed in the central area of the TP (2750–3750 m), and possess partial scales, a single pair of pharyngeal teeth and barbels. The HSG comprises six genera (*Gymnocypris*, *Oxygymnocypris*, *Schizopygopsis, Chuanchia*, *Platypharodon* and *Herzensteinia*), including 26 species and subspecies. They inhabit the middle and upper reaches of rivers (3750–4750 m), and possess few scales, pharyngeal teeth, and barbels. At the molecular level, it was suggested that the three groups of Schizothoracine fish belong to independent branches of phylogenetic tree based on a mtDNA *Cyt-b* marker [16,19]. Recently, phylogenetic reconstruction of mtDNA (including *Cyt-b*, *16SrRNA*, *COI* and *ND4*) and nuclear DNA *RAG2* gene [22] or mitochondrial genomes [23] has grouped together the three groups of Schizothoracine fish. However, He et al. [16,19] have suggested that Schizothoracine fish originated from a single clade of Barbinae. Conversely, Wang et al. [22] and Yonezawa et al. [23] suggested that Schizothoracine fish could be polyphyletic, with PG and SG+HSG possibly originating from two distinct clades of Barbinae. Generally, these results supported the idea for three groups of Schizothoracine that follow phenotypic evidences, like the scale loss, and using molecular markers. In addition, during the process of adaptation to tougher conditions such as lower temperature, longer freeze periods, and more limited food, investigators have generated a consensus that the SG with partial scales originated from one or more ancestral PG species with complete scales, and the HSG with few scales are the closest relatives or the most recent common ancestor, which includes the SG *Ptychobarbus*. Therefore, Schizothoracine fish are an ideal model to explore the molecular mechanisms linked to the scale loss associated with the environmental evolution of the TP. 

Past evidence has shown that ectodysplasin A (Eda) belongs to a tumor necrosis factor family ligand that is involved in the development of various structures derived from the ectoderm, including hair, teeth, sweat glands, feathers, armor plates, and scales [24]. The Eda protein contains four transmembrane region (TM) domains, a furin consensus cleavage site, a collagen-like domain (CL) and a tumor necrosis factor (TNF) domain [25]. Variations within those three Eda domains could result in hypohidrotic ectodermal dysplasia (HED) in humans [26,27]. The phenotypic characteristics associated with this disorder include sparse hair, abnormal or missing teeth, and an inability to sweat as a result of absent sweat glands. In mice, Srivastava et al. [28] observed that the tabby phenotype (characteristic hair defects, tooth abnormalities, and eccrine sweat gland morphology) is caused by a mutation in the *Eda* gene. Recent studies have shown that specific *Eda* orthologous genes have given rise to scales and armor plate phenotypes in fish. Harris et al. [29] screened mutant genes that have been demonstrated to be essential to the formation of adult skeletal structures (including scales) in zebrafish. Furthermore, the number of bony armor plates in three-spined sticklebacks has been shown to have decreased during the process of migration from marine to freshwater. Colosimo et al. [30] reported that compared to completely plated populations, low-plated populations harbor the same variations at the *Eda* locus. This result revealed that *Eda* evolution plays a critical role in determining the armor plate phenotype of the three-spined stickleback when exposed to similar ecological conditions. This interesting evolutionary phenomenon has been observed in vertebrates, including fish, birds, and humans [31,32,33,34,35]. The phenomenon is also observed in Schizothoracine fish. As a result of this study, we suggest that the evolution of *Eda* might be related to the changes associated with the ancient environment in the TP. We hypothesize that these changes might have given rise to the formation of various scale-related phenotypes and adaptations to different altitudes. 

The present study, we constructed phylogeny tree based on the complete coding sequences of the *Eda* gene of 51 Schizothoracine fish species and five Barbinae species, and identified sequence mutations and protein structures variations. Our aim was to investigate the molecular basis of scales loss in Schizothoracine fish, and to reveal the relationship between gradual scale loss in Schizothoracine fish and the three phased uplift events of TP. 

## 2. Results

### 2.1. Eda Genes of 51 Schizothoracine Species and Five Barbinae Species

**Figure 1:**
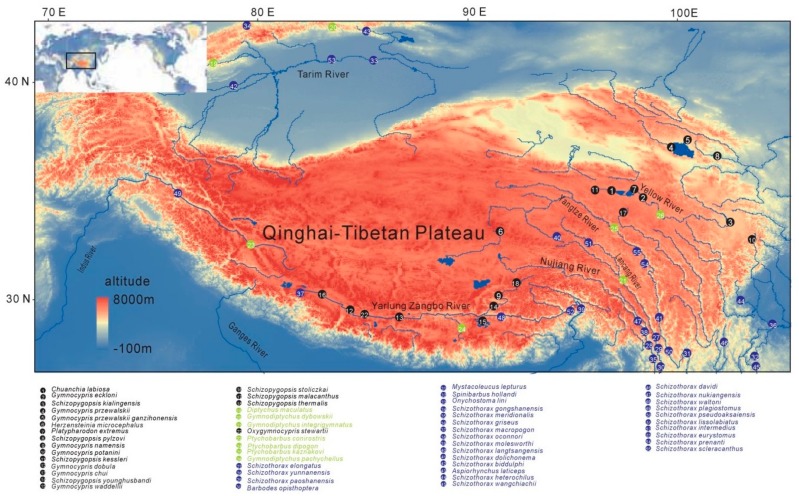
Geographical distribution of Schizothoracine fish samples on the Tibetan plateau (TP). The location where the Schizothoracine fish samples were collected is showd and the groups to which the samples belong are indicated with different colors. The highly specialized group (HSG) is in black, the specialized group (SG) is in green and the primitive (PG) in blue. The altitude ranges from 0 to 8000 m. The names of the main rivers in the TP are shown, including the Yangtze, Yellow, Yarlung Zangbo and Indus rivers. The big and small maps in this figure were generated using ArcGIS 10.2 (http://www.esri.com/).

We collected 141 samples from 51 Schizothoracine fish species and eight samples from five Barbinae fish species, distributed at different altitudes of the TP (Figure 1). A sequence of 1213 bp in length from *G. przewalskii Eda* cDNA was PCR amplified and the match of the sequenced hit with the *Eda* gene was verified via BLAST nucleotide. Specific primers (Table S1) were used to amplify the *Eda* cDNA of the remaining 51 Schizothoracine fish species (including PG, SG and HSG) and five Barbinae fish species. The *Eda* coding sequence (CDS) was observed to be 1059 bp in length in 19 HSG species, encoding a protein consisting of 352 amino acids (aa). Two scenarios in *Eda* CDS length was observed in seven SG species, one is 1059 bp, and the other is 1065 bp, which encode for 352 aa and 354 aa, respectively. Finally, the length of the *Eda* CDS (1083 bp in length) in 25 PG species is consistent with that of the five Barbinae species, and both encode a protein of 360 aa. 

### 2.2. Phylogenetic Reconstruction of the Eda Genes of Three Schizothoracine Groups

**Figure 2:**
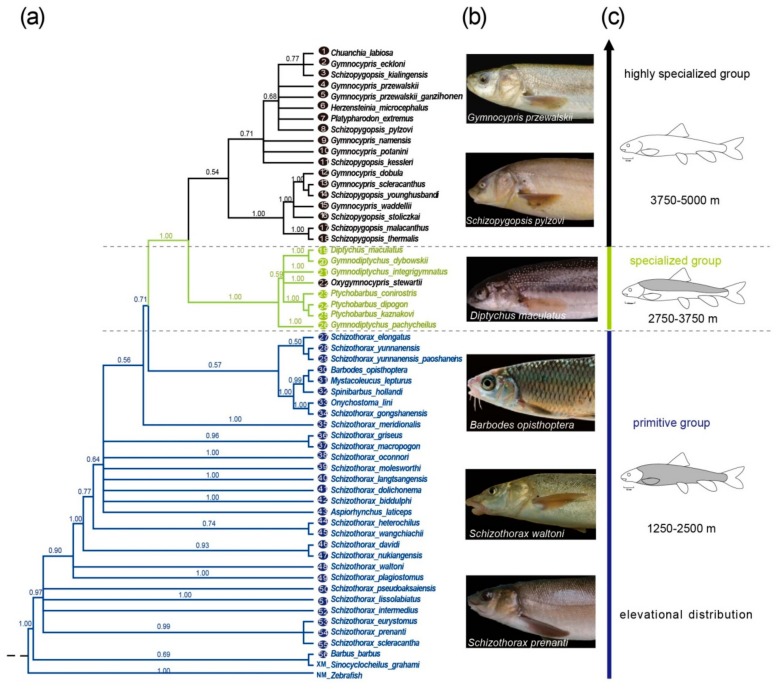
Phylogeny of the *Eda* gene in Schizothoracine fish on the TP. (**a**) Phylogenetic tree of the *Eda* gene of 58 fishes using the Bayesian analysis. The high specialized group (HSG) is indicated in black, the specialized group (SG) is in green and the primitive group (PG) is in blue, the HSG species were grouped into one clade, those of the SG species were gathered into another clade except for *Oxygymnocypris stewartii*. (**b**) The pictures of *Schizothorax prenanti*, *Schizothorax waltoni*, *Diptychus maculates*, *Gymnocypris przewalskii Schizopygopsis pylzovi* and *Barbodes opisthoptera*. (**c**) The straight line with the arrow in three colors represents the elevational distribution of the three groups within Schizothoracine and Barbinae fish species.

To analyze the relationships between the history of *Eda* sequence changes and the scale loss in Schizothoracine fish, we generated phylogenetic trees using the *Eda* CDS of the Schizothoracine fish and Barbinae fish species (Figure 2a). The Bayesian and maximum likelihood trees all suggested that the *Eda* sequences could be divided into two distinct clades, one clade containing the PG species, and the other clade comprising SG and HSG species (posterior probability of 0.71 in the Bayesian tree, bootstrap support of 99.7% and 83% in maximum likelihood trees). In the latter clade, differentiation between the SG and HSG species was observed (posterior probability of 1.00 in the Bayesian tree and bootstrap support of 99.7% in the maximum likelihood trees). The results also suggested that *Oxygymnocypris stewartii* is a transitioning species from the SG to the HSG. Additionally, as part of this study, we also observed a close relationship between Barbinae fish and the PG species. Combining the degree of scales (Figure 2b) and elevational distribution (Figure 2c), we found that the three distinct clades of Eda coincide with gradual scale loss from PG (complete scales) to SG (partial scales) to HSG (few scales), and elevational distribution gradually increasing from lower than 2750 m to 2750–3750 m to higher than 3750 m. These results suggested that *Eda* evolution is associated with scale loss and elevational distribution in Schizothoracine fish.

### 2.3. Multiple Sequence Alignment of the Eda Gene in Schizothoracine and Barbus Species

**Figure 3:**
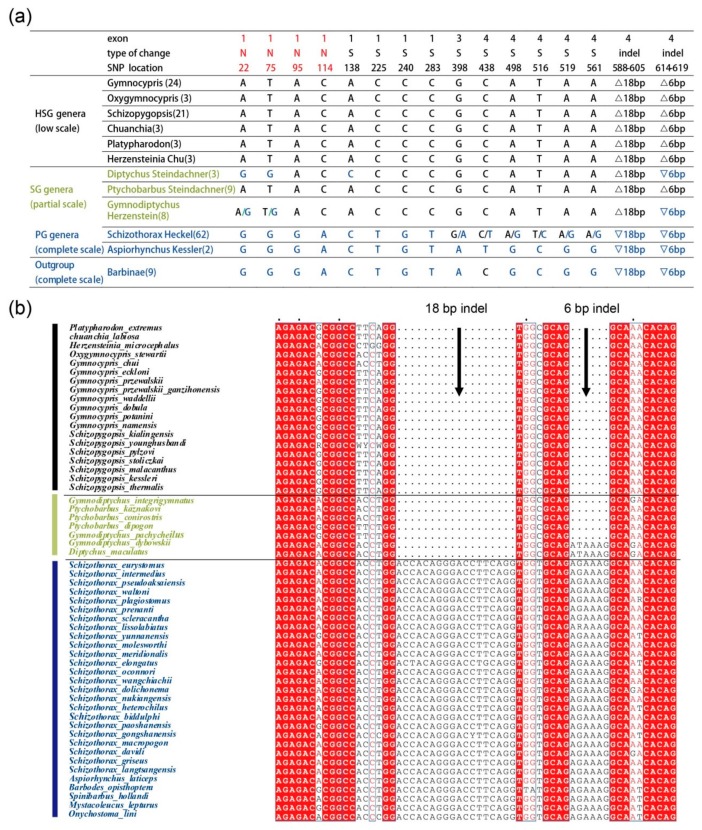
The alignment of *Eda* sequences in the PG, SG and HSG Schizothoracine fish. (**a**) According to alignment results generated by MUSCLE (http://www.ebi.ac.uk/Tools/msa/muscle/), the common 14 SNPs and two deletions were found in six genera of the HSG based on sequence comparison with the PG group. “N” pertains to nonsynonymous mutations, and “S” are the synonymous mutations. “△” represents deletions, and “▽” are insertion within the *Eda* coding sequences of Schizothoracine fish. All species in the genera are shown in the Table S2. (**b**) Alignment results of the partial variant of *Eda* exon 4 shows that the 18 bp and 6 bp deletions occurred only in the SG and HSG, and not in the PG.

To refine the position of the major locus, all the CDSs of the fish species were aligned using MUSCLE software (http://www.ebi.ac.uk/Tools/msa/muscle/). Following this alignment, we detected a total of 266 SNPs. By comparing the HSG and SG with PG group, we found the same 14 SNPs in 57 samples of the HSG groups belonging to 6 genera, 19 species (Figure 3a). The SNPs are in the ORF positions 22 (G > A), 75 (G > T), 95 (A > G), 114 (C > A), 138 (A > C), 225 (C > T), 240 (C > G), 283 (C > T), 398 (G > A), 438 (C > T), 498 (A > G), 516 (T > C), 519 (A > G), 561 (A > G), and all of these SNPs are located within exons 1 and 4, except for one that is situated in exon 3 (Figure 3a). In particular, four non-synonymous mutations, that is Thr8Ala, His25Gln, Asn32Ser and Asn38Lys caused by 22 (G > A), SNP 75 (G > T), SNP 95 (A > G) and SNP 114 (C > A) at *Eda* exon 1, may have driven potential changes in protein structure. Additionally, an 18 bp and a 6 bp indel in exon 4 of the *Eda* gene were observed in the 58 species (Figure 3b). Interestingly, the two indels were observed at the boundaries of the three Schizothoracine fish groups. By comparing with the *Eda* sequences from all members of the PG, the *Eda* sequences from two members of SG (*Diptychus maculates* and *Gymnodiptychus dybowskii)* harbored an 18 bp deletion. Additionally, the *Eda* sequences observed in the HSG contained 18 bp and 6 bp deletions at exon 4. In terms of scale coverage, the whole bodies of samples of *D. maculates* and *G. dybowskii* (apart from the abdomen) were covered with scales. The degree of scale coverage associated with the other five species was more comparable with the HSG species. These two ins/del mutations did not cause a shift in the reading frame, although these did change the number of repeats (G-X-Y)_n_ that comprise the collagen-like domain of the Eda protein. 

### 2.4. Homologous Sequence Alignment and Structure Prediction of the Eda Protein 

**Figure 4:**
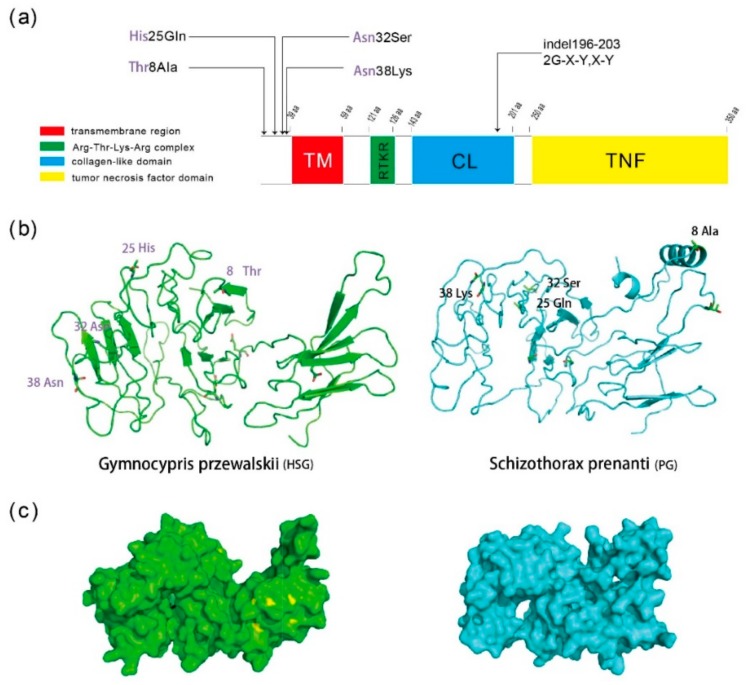
Amino acid and structure prediction of the Eda protein in Schizothoracine fish on the TP. (**a**) Schematic diagram of the Eda protein and the location of common non-synonymous mutations and two indels in the three groups of Schizothoracine fish. The Eda protein contains four main functional domains: a transmembrane domain (TM), an Arg-Thr-Lys-Arg complex (RTKR), a collagen-like domain (CL) and a tumor necrosis factor domain (TNF). The length of each domain is indicated by a scale plate ranging from 20 aa to 100 aa. The four consistent sites are shown in the three groups of Schizothoracine fish, i.e., the high specialized group (HSG), the specialized group (SG) and the primitive group (PG). Only two motifs, namely G-X-Y and X-Y were mapped to the key domain, CL of the Eda protein. (**b**,**c**) are the secondary and tertiary structures of the Eda protein of HSG and PG Schizothoracine fishes. The common non-synonymous mutations, including Thr8Ala, His25Gln, Asn32Ser and Asn38Lys are marked in the secondary structure of the Eda protein of HSG and PG Schizothoracine fishes.

To further test whether the protein domain in which the indels occur is highly conserved amongst teleosts, we compared all *Eda* coding sequences of teleost that are available in NCBI and Ensembl. We aligned the amino acid sequences of Schizothoracine fishes with the Eda protein sequences of other teleosts using COBALT. The results showed that the two G-X-Y and one XY motif deletion (encoded by the 18 bp and 6 bp deletion) were detected exclusively in HSG Schizothoracine fishes, and the two G-X-Y motif deletion was observed in *G. dybowskii* and *Diptychus maculatus*, and only the X or XY motif deletion was identified in the other teleost groups (Figure S1). Considering that the HSG Schizothoracine fishes shared the same 14 SNPs and two deletions compare to the PG Schizothoracine fishes, the same genotype might be associated with scale loss in the HSG species. In particular, four non-synonymous mutations and two indels can cause significant differences in the Eda structures of the HSG and PG species. However, only two indel sites were mapped to the collagen-like domain of the Eda protein, four non-synonymous mutations were not mapped to the four key domains of the Eda protein (Figure 4a), but this does not mean that these sites have no effect on the overall structure of the Eda protein. To decipher the potential functional impact of the non-synonymous mutations and indels, we generated the secondary structure of Eda protein in the HSG and PG Schizothoracine fishes (Figure 4b,c). Distinct differences in the secondary structure and tertiary structure of the HSG and PG Eda protein were observed. For example, compared to the Eda of HSG, a spiral structure was observed in the PG Schizothoracine fishes. Moreover, four non-synonymous mutations, including Thr8Ala, His25Gln, Asn32Ser and Asn38Lys, may have contributed to the secondary structure differences in the Eda protein of the HSG and PG Schizothoracine fishes (Figure 4b,c). 

## 3. Discussion

Schizothoracine fish are the main fish species distributed across the TP. In conjunction with uplift of the TP, Schizothoracine experienced three dramatic environmental changes. Based on the altitude of their habitat, their scale varies from whole body coverage, to local body coverage, to nearly no scale coverage over the entire body (Figure 1). Using scale traits as criterion, the Schizothoracine fish have been classified to three different biological patterns (primitive, specialized, and highly specialized groups), which in turn correspond to their altitude specific distribution. The phenotype variations involving scales support the common theory in evolutionary biology that organisms will go through evident phenotypic changes during their adaptation to new ecological niches especially caused by geological vicissitude [36,37,38,39]. Scale loss in the Schizothoracine fish with increasing elevation could chiefly be the result of long term adaptation to their environment. The higher the altitude, the lower the temperature, and the stronger the solar radiation on the TP. To avoid UV light and cold, the fish is remains in the cave during the cold season for nearly half a year and spends most time of the other period at the higher altitudes. This burrowing life styles leads to the scale degeneration in Schizothoracine fish [11] which is similar to that of other burrowing fish such as *Sinocyclocheilus* cavefish [40]. In the three types of *Sinocyclocheilus*, including the surface-dwelling *S. grahami* (*Sg*), the semi-cave-dwelling *S. rhinocerous* (*Sr*), and the cave-restricted *S. anshuiensis* (*Sa*), Yang et al. [40] found two copies of ectodysplasin a receptor (namely *Edar1* and *Edar2*). The Edar1 of the three Sinocyclocheilus species has deletions in its signal peptide and partial extracellular domains Yang et al. [40] discovered that the signal peptide and partial extracellular regions of Edar2 in *Sa* were totally deleted when compared with *Sg* and *Sr*. The deficiency of this domain in *Sa* may lead to disruption in guiding the transport of the Edar protein across the membrane, thus generating fewer scales on the skin surface of *Sa*. Eda signaling is mediated by Eda, Edar and Edar associated adapter protein, which are involved in skin appendage development in vertebrates from fish to humans [24]. Scale loss in Sinocyclocheilus is associated with the *Edar* gene [40]. The present study detected interesting results regarding *Eda* gene, which coincide with Schizothoracine fish scale development. 

The *Eda* gene phylogenetic tree (Figure 2) suggested that three distinct clades consisting of the PG, SG, and HSG (apart from *Oxygymnocypris stewartii*, which belongs to the SG), which is highly consistent with the phenotypic clusters and the the mitochondrial *cytb* gene [16,19], but differs from the phylogeny produced with mtDNA (including *Cyt-b*, *16SrRNA*, *COI*, and *ND4*) and nuclear DNA *RAG2* gene [22] or mitochondrial genomes [23]. According to the dendrogram of Wang et al. [22] and Yonezawa et al. [23], the PG was evolved from a clade of Barbinae (*Onychostoma lini*, *Spinibarbus hollandi*, etc.), and the SG and HSG had arisen from another clade of Barbinae (*Barbaus barbus*, *Scaphiodonichthys acanthopterus and Luciobarbus capito*). In other words, the SG and HSG belong to another clade that included *Barbaus barbus*, *Scaphiodonichthys acanthopterus*, and *Luciobarbus capito*. However, the *Eda* phylogenetic tree revealed that the SG and HSG are two independent clades, and *Barbaus barbus* is grouped together with the PG and other Barbinae species. These results support the view that the *Eda* gene may be responsible for the involution of scales in Schizothoracine fish, but do not represent their origin and differentiation. 

*Eda* plays a vital role in scale development [29], and evidence suggests that the evolution of the three main clades, and the corresponding relationship between scale loss and species differentiation may be reflected through comparative analysis of this gene. Moreover, 14 SNPs and 18 bp and 6 bp indels can be used to distinguish between the three groups. Each of the PG species and the *Barbinae* fish, and two of the SG species share one genotype of 14 SNPs and the 18 bp ins mutation in *Eda* exon 4. The HSG species harbor another genotype of 14 SNPs and both 18 bp and 6 bp deletions in *Eda* exon 4. These results suggest that the PG species might have adapted to the 1250–2500 m altitude environment following the first uplift event of the TP, with their scales (apart from scale size) showing similarities to the *Barbinae* fish (Figure 2). Interestingly, we detected an 18 bp deletion in the *Eda* of *D. maculatus* and *G. dybowskii*, and two deletions (18 bp and 6 bp in size) in another five SG species and all of the HSG species that were analyzed. As the member of the SG-related subspecies, *D. maculatus* and *G. dybowskii* were collected from lowland locations (<2100 m) in the Xinjiang Province, respectively. We argue that the lower altitude may explain why these species are covered with more scales than the other SG species. We thus propose that the gradual scale loss in Schizothoracine fish may be associated with the altitude of their habitat. 

The same variations were observed in the *Eda* locus in HSG Schizothoracine fishes, and only two variant residues were located in the collagen-like domain of the Eda protein. Furthermore, both the 18 bp and 6 bp ins/del mutations resulted in changes in the number of signature repeats [(G-X-Y)_19_ < (G-X-Y)_17_] and the XY amino acid located at the CL domain of the Eda protein. The collagen domain is the main functional domain associated with fibrous collagen proteins in vertebrates, and the associated gene is important for the development of skin, bones, tendons, cartilage, blood vessels, and teeth [41,42]. The collagen-like domain of Eda serves a similar function to the collagen proteins, and two or more Eda monomers comprise the trimers that potentiate stimulation associated with Edar signaling [43,44,45]. Finally, the proteoglycan-binding domain, which is encoded by exon 4, restricts Eda diffusion in tissues following its release in its soluble form [45]. The mutations are associated with X-linked HED and accumulate in the TNF regions. However, several in-frame deletions have been observed in HED patients, indicating that the collagen-like domain also harbors other important functions that have not been elucidated to date. The domain comprises 19 G-X-Y repeats in two neighboring stretches [28]. Deletion of two or four G-X-Y repeats has no effect on the functional TNF domain in Eda syndrome [44,46,47]. Therefore, the analysis further supported the foregoing conjecture. 

We demonstrate that the protein domain in which the indels occur is relatively conserved amongst 17 teleosts for which *Eda* sequences are available in NCBI and ENSMBL by using COBALT (www.ncbi.nlm.nih.gov/tools/cobalt/). The conservation of the domain in teleosts with complete scales demonstrates that it is essential for proper Eda function. In addition, the 18 bp and 6 bp deletions may be reponsible for scale loss in specialized Schizothoracine fishes. The secondary and tertiary structures of the Eda protein significantly differ between HSG and PG Schizothoracine fishes, and thus the function of Eda in relation to scale may also be affected. The function of the collagen-like domain of the Eda protein in fish scale development thus requires further investigation. Similar studies have been performed in mouse and stickleback models [30]. Direct confirmation that the changing levels of Eda signaling result in altered plate development has been observed in sticklebacks. Colosimo et al. [30] injected single-celled embryos from low-plated parents with full-length mouse *Eda-A1* cDNA. The numbers of armor plates from the transgenic fish were then compared to wild-type fish. Their results confirmed that Eda signaling triggers of lateral plate formation. 

In conclusion, with the three dramatic uplift events of the TP, the fish must adapt themselves to the changes in the environment, which include temperature, ice periods, limited food. Thus, only the fittest fish adapt to the severe environments can survive. Based on natural selection, the Schizothoracine have differentiated into the HG, SG and HSG. In terms of lifestyles, the few scales HSG adapted to the burrowing life, partial scale SG fish have adapted to the semi-burrowing life, and the completely scales PG live in the warm water. During cold periods, to avoid the discomfort of low temperatures and to capture more food, fish must live in the bottom part or even in the narrow stone crevices of the river and/or lakes. in addition, the higher the altitude, the longer the cold season, the fish with fewer scales can survive in this condition [11,40]. Therefore, the HSG species share the characteristic *Eda* sequence changes that are related to having few scales compared to the PG and SG, allowing them adapt to the severe environment. 

## 4. Materials and Methods

### 4.1. Fish Sampling, RNA and Genomic DNA Extraction

All animal experiments were approved by the Animal Care and Use Committees of the Northwest Institute of Plateau Biology, Chinese Academy of Sciences. All experiments and trials were conducted in accordance with the laws and regulations controlling experiments and procedures in live animals. Fish samples (Schizothoracine fish and Barbinae fish) were sampled from three groups distributed on the TP (Figure 1, Table S2). All fish samples (fins and muscle) were preserved in 75% ethyl alcohol and liquid nitrogen. Photographic data of each fish samples were generated using a Canon EOS 600D camera. This was performed on live fish species or specimens from the Museum of Qinghai-Tibetan Plateau (Xining, China). RNA and DNA isolation was performed using TRIzol reagent (Invitrogen, Carlsbad, CA, USA) and genomic DNA isolation kit (QiaGene, Frankfurt, Germany) according to the manufacturer’s protocol. 

### 4.2. Eda Sequencing and Assembly Strategy

cDNA template preparation was performed as follows: 2 μg of RNA and 0. 5 μg of Oligo(dT)_16_ were incubated at 70 °C for 5 min. Following a 2-min ice-incubation stage, the mixture was reverse transcribed using 200 U of M-MLV reverse transcriptase, 5× buffer, 25 U of RNase, and 0.8 mM dNTPs in a total volume of 25 μL. Extension was performed for 1 h at 42 °C. We surveyed the *Gymnocypris przewalskii* transcriptome dataset [48] to identify a potential *Eda* sequence using zebrafish *Eda* sequence as a query. Next, we designed the gene-specific primers using soft of Primer Premier 5.0 software (Premier, QC, Canada, Table S1) to clone the *Eda* cDNA sequences from Schizothoracine fish samples with cDNA. 

For the remaining Schizothoracine fish and Barbinae fish samples with only genomic DNA, we continued to design gene-specific primers (Table S1) to facilitate cloning of exons 1 to 8 of the *Eda* gene and assemble by overlap. The specificity of all primers were tested by NCBI BLAST. The following procedure was used to amplify the desired sequences: pre-denaturation was performed at 94 °C for 5 min; then 35 cycles of 94 °C for 30 s, specific annealing temperature (Table S1) for 30 s, 72 °C for 30 s; and a final extension step at 72 °C for 7 min using a Veriti Thermal Cycler PCR system (Applied Biosystems, Inc., Carlsbad, CA, USA). All of the PCR products that were generated were analyzed using a 1% EtBr-agarose gel, and products were visualized and photographed. The amplicons were excised and extracted with a DNA Gel Extraction Kit (Sangon, Shanghai, China). The amplicon sequences were cloned into the pGEM-T easy vector (Promega, Madison, WI, USA), sequenced using an ABI3730XL sequencer (Beijing Tianyi Huiyuan Bioscience and Technology Inc., Beijing, China), and analyzed using DNAStar (DNASTAR, Madison, WI, USA) and Clone Manager Professional Suite 8 (Scientific & Educational Software, Denver, CO, USA). 

### 4.3. Multiple Sequence Alignments and Phylogenetic Analysis

All *Eda* CDSs sequences of Schizothoracine fishes were aligned with MUSCLE (http://www.ebi.ac.uk/Tools/msa/muscle/), and Eda protein sequences of Schizothoracine fishes and other teleosts obtained from NCBI and Ensembl were compared with the online version of COBALT. 17 species of Eda protein sequences were used in the analysis, we obtained five sequences including those of *Gymnocypris przewalskii*, *Ptychobarbus dipogon*, *Diptychus maculatus*, *Schizothorax prenanti*, *Spinibarbus hollandi*, 12 sequences from NCBI and Ensembl, including that of *stickleback*(ENSGACP00000024202), *medaka* (ENSORLP00000008186), *Danio rerio* (NP_001108537.1), *Scleropages formosus* (XP_018602147.1), *Clupea harengus* (XP_012674999.1), *Stegastes partitus* (XP_008279534.1), *Kryptolebias marmoratus* (XP_017282113.1), *Cyprinodon variegates* (XP_015243980.1), *Nothobranchius furzeri* (SBP63202.1), *Xiphophorus maculates* (XP_005795568.1), *Oryzias latipes* (XP_004073413.1), and *Oreochromis niloticus* (XP_003445927.1). The website of COBALT is www.ncbi.nlm.nih.gov/tools/cobalt/. MrBayes and RAxML trees were constructed using CIPRES (https://www.phylo.org/portal2/). Bayesian inference (BI) was conducted in MrBayes v.3.2.6 [49] with unlinked branch lengths and a partitioning scheme suggested by PartitionFinder. Two independent runs of one million generations, each comprising four Markov chain Monte Carlo (MCMC) chains, were sampled every 1000 generations with a 25% burn-in. We checked the average standard deviation of split frequencies for assessing convergence by <0.01. The posterior probabilities (PP) indicating support values for each branch were also estimated. RAxML analyses were performed using the graphical front-end RAxML GUI v1.3.1 [50] with a GTRGAMMAI model for each partition identified by Partitionfinder. Search for the most likely ML tree and calculation of nodal bootstrap support (BS) were carried out using the “ML + rapid bootstrap” option with nonparametric 1000 bootstrap replicates. 

### 4.4. Eda Protein Structure Analysis

To better understand the potential functional divergence of *Eda* gene in Schizothoracine, we selected two species, *Schizothorax prenanti* and *Gymnocypris przewalskii* as the representative species from the PG and HSG to predict and compare their secondary and tertiary structures of protein. Due to the lack of crystal structure of Eda protein in any Schizothoracine, we submit the amino acids sequences of above two fish species to the I-TASSER online server (https://zhanglab.ccmb.med.umich.edu/I-TASSER/) for de novo modeling according to default parameters. Next, we used the cartoon and surface models in PyMOL software [51] to exhibit the Eda protein structure following the manual, respectively. At last, we mapped the nonsynonymous mutations and positive selection sites onto the 3D protein structure of Eda protein using PyMOL for better visualization.

## Supplementary Materials

It can be found at: http://www.mdpi.com/1422-0067/19/10/2953/s1.

## Data Accessibility

DNA sequences are deposited to the Genbank under Accessions numbers: KT239680-KT239734.
