# Supplementary material for: Adaptive Evolution of the Eda Gene and Scales Loss in Schizothoracine Fishes in Response to Uplift of the Tibetan Plateau
Source: Int J Mol Sci. 2018 Sep 27;19(10):2953. doi: 10.3390/ijms19102953 (PMC6213870; doi:10.3390/ijms19102953)
Supplement: Supplementary file 1 [file ijms-19-02953-s001.zip › supplementary/Fig.S1-..pdf]

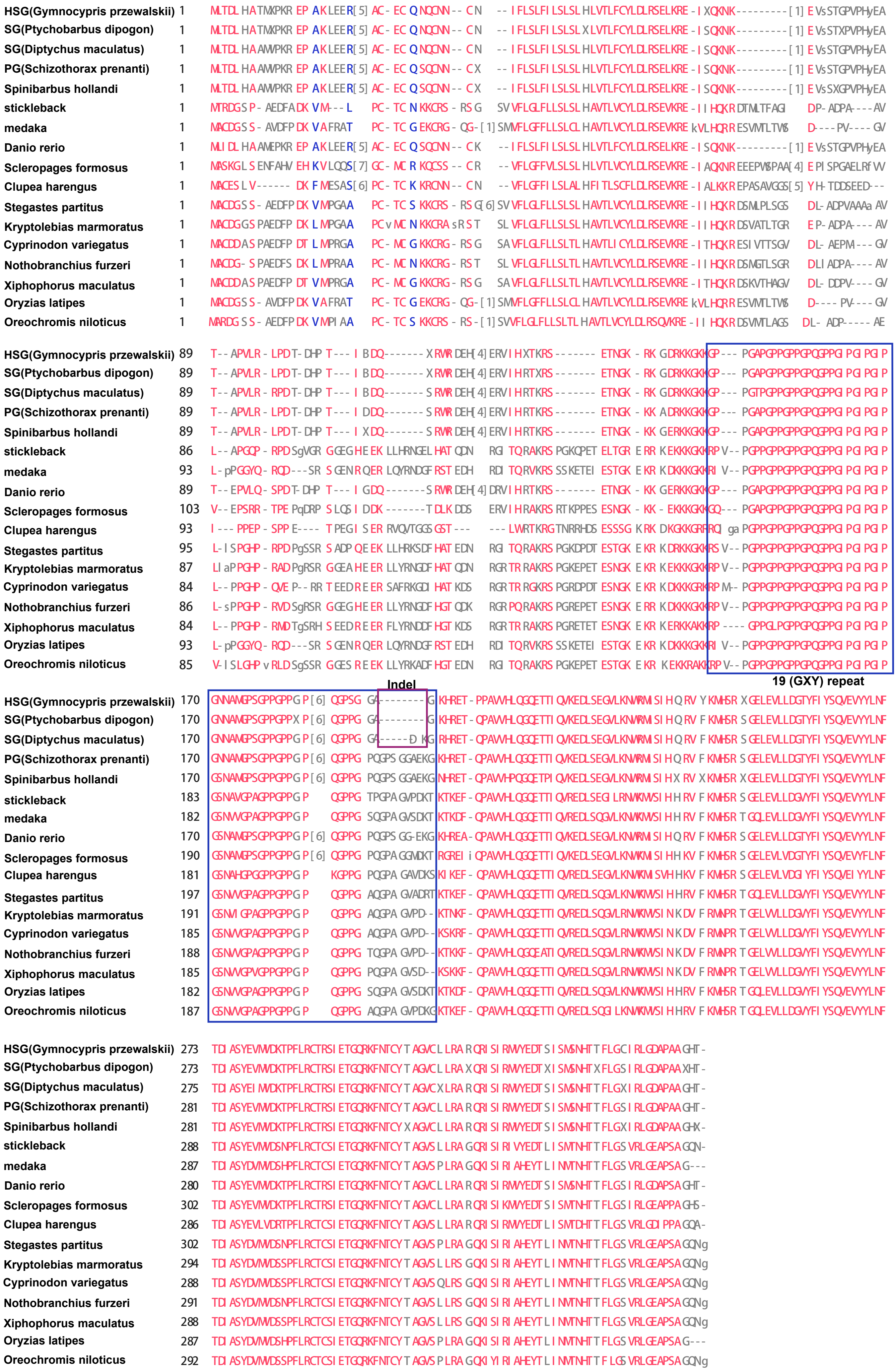
HSG(Gymnocypris przewalskii)
SG(Ptychobarbus dipogon)
SG(Diptychus maculatus)
PG(Schizothorax prenanti)
Spinibarbus hollandi
stickleback
medaka
Danio rerio
Scleropages formosus
Clupea harengus
Stegastes partitus
Kryptolebias marmoratus
Cyprinodon variegatus
Nothobranchius furzeri
Xiphophorus maculatus
Oryzias latipes
Oreochromis niloticus
Indel
19 (GXY) repeat
