# Supplementary material for: Adaptive Evolution of the Eda Gene and Scales Loss in Schizothoracine Fishes in Response to Uplift of the Tibetan Plateau
Source: Int J Mol Sci. 2018 Sep 27;19(10):2953. doi: 10.3390/ijms19102953 (PMC6213870; doi:10.3390/ijms19102953)
Supplement: Supplementary file 1 [file ijms-19-02953-s001.zip › supplementary/table S1.pdf]

Table S1. Sequences of the PCR primers for amplifying *Eda* gene.

Eda Gene	Oligonucleotides for PCR, 5′ to 3′		Annealing Temperature	Amplicon Size, bp
cDNA	F: AAGTCGCTGAGAGGACGAGGAAGTT	R: CGACCCTATCAGACACACACATCACG	58 ℃	1103-1127
Exon 1	F: AAGTCGCTGAGAGGACGAGGAAGTT	R: GGACACCGACCACCCAACAATAGA	67 ℃	351
Exon 2	F: GAGCAGATGGAGGGATGAACA	R: ATTATTGGGCTATGTTGTGCG	58 ℃	327
Exon 3 and 4	F: GTCATTATCGGTTTGGCTAC	R: AAACTCTTCCCTTCAGCAA	52 ℃	670-700
Exon 5and 6	F: GCAAAAACATTTCTCTTCTCTGACA	R: ACTGGCTGTTGTAAATGTTCTTTGT	60 ℃	850-880
Exon 7 and 8	F: TCACTGTGTTTTAACTGTGCCGCTG	R: CGACCCTATCAGACACACACATCACG	68 ℃	649-669
Target site	F: TTGTTTTGCTTCTCATCAGTTG	R: TTTGCTCTGCTGCTTCACTC	58 ℃	357
