# Supplementary material for: Adaptive Evolution of the Eda Gene and Scales Loss in Schizothoracine Fishes in Response to Uplift of the Tibetan Plateau
Source: Int J Mol Sci. 2018 Sep 27;19(10):2953. doi: 10.3390/ijms19102953 (PMC6213870; doi:10.3390/ijms19102953)
Supplement: Supplementary file 1 [file ijms-19-02953-s001.zip › supplementary/table s2.pdf]

Table S2. Sample and identification information pertaining to *Eda* sequences used in this study.

Group	Species	Collecting Site	Catchment	Drainage System	Number of Samples	Accession Number
Barbinae	*Barbodes opisthoptera*	Baoshan, Yunnan	Nujiang River	Nujiang River	2	KT239731
	Spinibarbus hollandi	Lijiang, Guangxi	Lijiang River	Zhujiang River	2	KT239732
	Mystacoleucus lepturus	Xishuangbanna Yunnan	Lancang River	Lancang River	2	KT239733
	Onychostoma lini	Rong'an, Guangxi	Zhujiang River	Zhujiang River	2	KT239734
Primitive groups	*Schizothorax eurystomus*	Yecheng , Xinjiang	Tizinafu River	Tarim River	3	KT239706
	Schizothorax intermedius	Yecheng, Xinjiang	Tizinafu River	Tarim River	3	KT239707
	Schizothorax prenanti	ABa, Sichuan	Dadu River	Yangtze River	3	KT239711
	Schizothorax scleracantha	Ganzi, Sichuan	Jinsha River	Yangtze River	2	KT239712
	Schizothorax pseudoaksaiensis	Xinyuan, Xinjiang	Kunnes River	Yili River	3	KT239708
	Schizothorax waltoni	Lhasa ,Tibet	Lhasa River	Yarlung Zangbo River	3	KT239709
	Schizothorax plagiostomus	Gar,Tibet	Gar River	Indus River	2	KT239710
	Schizothorax molesworthi	Medog, Tibet	Yarlung Zangbo River	Yarlung Zangbo River	2	KT239715
	Schizothorax meridionalis	Tengchong, Yunnan	Longchuan River	Irrawaddy River	2	KT239716
	Schizothorax elongatus	Yingjiang, Yunnan	Irrawaddy River	Irrawaddy River	3	KT239717
	Schizothorax oconnori	Lhasa ,Tibet	Lhasa River	Yarlung Zangbo River	3	KT239718
	Schizothorax wangchiachii	Shigu, Yunnan	Jinsha River	Yangtze River	3	KT239719
	Schizothorax dolichonema	Yushu, Qinghai	Jinsha River	Yangtze River	2	KT239720
	Schizothorax nukiangensis	Zuogong,Tibet	Nujiang River	Nujiang River	3	KT239721
	Schizothorax heterochilus	Ya'an Sichuan	Qingyi River	Yangtze River	3	KT239722
	Schizothorax lissolabiatus	Nangqian, Qinghai	Zhaqu River	Lancang River	3	KT239713
	Schizothorax biddulphi	Wuqia, Xinjiang	Tarim River	Tarim River	3	KT239723
	Schizothorax yunnanensis	Tengchong, Yunnan	Lancang River	Lancang River	2	KT239714
	Schizothorax yunnanensis paoshanensis	Baoshan, Yunnan	Baoshan east river	Nujiang River	2	KT239724
	Schizothorax_gongshanensis	Gongshan, Yunnan	Nujiang River	Nujiang River	2	KT239725
	Schizothorax macropogon	Xigaze, Tibet	Yarlung Zangbo River	Yarlung Zangbo River	3	KT239726
	Schizothorax griseus	Guiyang, Guizhou	Wujiang River	Yangtze River	2	KT239728
	Schizothorax davidi	Wudu, Sichuan	Jialing River	Yangtze River	3	KT239727
	Schizothorax langtsangensis	Zadoi, Qinghai	Zhaqu River	Lancang River	2	KT239729
	Aspiorhynchus laticeps	Bazhou, Xinjiang	Bosten Lake	Tarim River	2	KT239730

Specialized groups	*Gymnodiptychus dybowskii*	Xinyuan, Xinjiang	Kunnes River	Yili River	3	KT239704
	Diptychus maculatus	Akeqi Xinjiang	Akesu River	Tarim River	3	KT239705
	Ptychobarbus conirostris	Gar,Tibet	Gar River	Indus River	3	KT239701
	Gymnodiptychus integrigymnatus	Tengchong, Yunnan	Longchuan River	Irrawaddy River	2	KT239699
	Ptychobarbus dipogon	Xigaze, Tibet	Yarlung Zangbo River	Yarlung Zangbo River	3	KT239702
	Ptychobarbus kaznakovi	Yushu, Qinghai	Jinsha River	Yangtze River	3	KT239700
	Gymnodiptychus pachycheilus	Jiuzhi, Qinghai	Yellow River	Yellow River	3	KT239703
Highly specialized groups	*Platypharodon extremus*	Maduo, Qinghai	Gyaring Lake	Yellow River	3	KT239680
	chuanchia labiosa	Maduo, Qinghai	Gyaring Lake	Yellow River	3	KT239681
	Herzensteinia microcephalus	Tanggula Shan, Qinghai	Tuotuo River	Yangtze River	3	KT239682
	Oxygymnocypris stewartii	Xigaze, Tibet	Yarlung Zangbo	Yarlung Zangbo River	3	KT239683
	Gymnocypris chui	Lazi, Tibet	Lange lake	Indus River	3	KT239684
	Gymnocypris namensis	Lhasa ,Tibet	Namsto Lake	Namsto Lake	3	KT239691
	Gymnocypris eckloni	Maduo, Qinghai	Gyaring Lake	Yellow River	3	KT239685
	Gymnocypris przewalskii	Gonghe, Qinghai	Qinghai Lake	Qinghai Lake	3	KT239686
	Gymnocypris przewalskii ganzihonensis	Haiyan, Qinghai	Ganzi River	Qinghai Lake	3	KT239687
	Gymnocypris waddellii	Langkazi, Tibet	Yamzho lake	Yamzho lake	3	KT239688
	Gymnocypris dobula	Xigaze,Tibet	Peiku lake	Peiku lake	3	KT239689
	Gymnocypris potanini	Songpan, Sichuan	Minjiang River	Yangtze River	3	KT239690
	Schizopygopsis kialingensis	Diebu, Gansu	Bailong River	Yangtze River	3	KT239692
	Schizopygopsis younghusbandi	Lhasa ,Tibet	Yarlung Zangbo	Yarlung Zangbo River	3	KT239693
	Schizopygopsis pylzovi	Haiyan, Qinghai	Yellow River	Yellow River	3	KT239694
	Schizopygopsis stoliczkai	Ali, Tibet	Shiquan	Indus River	3	KT239695
	Schizopygopsis malacanthus	Yushu, Qinghai	Tongtian River	Yangtze River	3	KT239696
	Schizopygopsis kessleri	Dulan, Qinghai	Dadu River	Yangtze River	3	KT239697
	Schizopygopsis thermalis	Naqu, Tibet	Nujiang River	Nujiang River	3	KT239698
